# ICSI in non-male factor infertility patients does not alter metabolomic signature in sibling embryos as evidenced by sensitivity enhanced nuclear magnetic resonance (NMR) spectroscopy

**DOI:** 10.1371/journal.pone.0273321

**Published:** 2022-09-23

**Authors:** Ameya Jijo, Aswathi Cheredath, Shubhashree Uppangala, Vani Lakshmi R., David Joseph, Huidrom Yaiphaba Meitei, Gitanjali Asampille, Pratap Kumar, Nagana Gowda G. A., Guruprasad Kalthur, Borut Kovacic, Satish Kumar Adiga

**Affiliations:** 1 Department of Reproductive Science, Kasturba Medical College, Manipal Academy of Higher Education, Manipal, India; 2 Department of Data Science, Prasanna School of Public Health, Manipal Academy of Higher Education, Manipal, India; 3 NMR Research Centre, Indian Institute of Science, Bangalore, India; 4 Department of Reproductive Medicine and Surgery, Kasturba Medical College, Manipal Academy of Higher Education, Manipal, India; 5 Northwest Metabolomics Research Center, Anesthesiology and Pain Medicine, University of Washington, Seattle, WA, United States of America; 6 Department of Reproductive Medicine, University Medical Centre Maribor, Maribor, Slovenia; University of Florida, UNITED STATES

## Abstract

Intracytoplasmic sperm injection (ICSI) was developed to overcome male factor infertility, however, there recently has been an increasing trend in ICSI usage irrespective of the etiology, demonstrating an overuse of this insemination technique. There is a limited knowledge on the behaviour of ICSI derived embryos in non-male factor infertility patients. Metabolomic assessment of preimplantation embryos in conjunction with morphological evaluation can provide better understanding of embryonic behaviour. Hence, this study was undertaken to explore if there are any metabolomic differences between IVF and ICSI derived sibling day-5 blastocysts from non-male factor infertility patients. This prospective study included nineteen couples with non-male factor infertility undergoing Assisted Reproductive Technology. The sibling oocytes retrieved from each patient were randomly assigned to two groups and inseminated either by IVF or ICSI. Spent culture media (SCM) in which embryos were cultured up to day 5 were collected and investigated using sensitivity enhanced NMR based metabolite profiling utilizing high resolution (800 MHz) NMR equipped with cryogenically cooled micro-coil (1.7 mm) probe. The metabolomic signature between IVF and ICSI derived sibling blastocysts was assessed. A significant reduction in the concentrations of pyruvate, citrate, glucose and lysine were observed in both IVF and ICSI sibling embryos compared to medium control (P< 0.05–0.001). Further, histidine and valine level was found lower in ICSI embryos compared to medium control (P<0.05) during 96 hours of *in vitro* culture. Notably, between IVF and ICSI SCM, no significant difference in the concentration of the metabolites was found. Our results suggest that ICSI in non-male factor does not alter the SCM metabolomic signature during 96 hours of embryonic development.

## Introduction

Non-male factor infertility conditions constitute approximately half of all infertility cases [1] and usually conventional in vitro fertilization (IVF) is recommended treatment in those conditions, particularly in tubal and idiopathic infertility. Intracytoplasmic sperm injection (ICSI) was developed to overcome male factor infertility, however, there recently has been an increasing trend in ICSI usage irrespective of the etiology, demonstrating an overuse of this insemination technique [2–5].

One reason to use ICSI in normo-responding patients to controlled ovarian stimulation (COS) with non-male factor conditions is due to an irrational fear of total fertilization failure [6]. Higher fertilization rate achieved with ICSI compared to IVF, and consequently an increased number of developed embryos resulting in a higher cumulative pregnancy rate, is an additional argument for preferring the use of ICSI as insemination method. However, the efficacy of ICSI in improving fertilization and avoiding the total fertilization failure in non-male factor patients is contradictory [7–10]. Similarly, live birth rates are not always higher in ICSI compared to IVF cycles of non-male factor patients [7,10–15]. Further, ICSI was not found superior to conventional IVF in non-male factor infertility with respect to preimplantation genetic testing for aneuploidies as both IVF and ICSI techniques generated equal numbers of euploid blastocysts [16].

Apart from the contradictory reports on the effectiveness of ICSI in non–male factor infertility, there are several identified risks associated with the technique such as the injection of polyvinyl pyrrolidone (PVP) exposed sperm, variations in temperature and time during ICSI, mechanical and osmotic trauma associated with the injection and suction, meiotic spindle damage and somatic cell DNA contamination [17–20]. Though, earlier few studies have failed to observe a relationship between any increase in the genetic [21] and epigenetic [22] abnormalities in ICSI born children, existing evidence indicates that children conceived through ICSI have an increased risk of chromosomal abnormalities [23], particularly those affecting sex chromosomes, compared with naturally conceived children [24,25]. ICSI offspring may experience poorer reproductive and metabolic health than spontaneously conceived children [26–30].

Considering all these facts, there is a need to thoroughly understand the embryonic behavior in relation to the mode of insemination in non-male factor infertile patients. While embryo morphology or morphokinetic evaluation can help in quick selection of embryos, it cannot precisely predict the embryo developmental capacity. Embryo metabolomics is an alternative and sensitive method that provides better insight in metabolic physiology and behavior of human embryos [31,32]. A recently published study looked into the metabolic profile of *in vitro* derived human embryos and found that mode of fertilization does not impair the embryo metabolism in moderate male factor infertility patients [33]. This group used ultramicrofluorometric assays and reverse-phase high performance liquid chromatography (HPLC) technique to measure the depletion of glucose, pyruvate, lactate and 18 amino acids in spent culture media.

The growing field of metabolomics promises immense opportunities for the investigation of cellular metabolism and viability using biological mixtures. It involves quantitative analysis of a large number of small molecules (MW ~1000 Da) in biological specimens in one-step. Application of metabolomics to human embryos has provided alternative, adjunctive technologies to understand the metabolic physiology and behavior [31,32,34]. Analytical techniques are pivotal to the metabolomics field [35,36] and nuclear magnetic resonance (NMR) spectroscopy is a powerful analytical technology that offers metabolic profiling of embryos non-invasively and with minimal sample preparation procedure [37]. In particular, NMR technology that combines high magnetic fields with cryogenically cooled micro-coil probes has emerged as a highly sensitive analytical platform for analysis of metabolites using a small quantity of samples such as the embryo SCM. Depending on the probe and sample tube used, the standard NMR profiling may require up to 600 μL of sample [37] whereas, embryo SCM may have approximately 20–30 μL, which is less compared to other somatic cell SCM profiling [38]. Furthermore, small volume metabolomics is common when *in vivo* brain metabolomics is performed using small animal models where ultra-sensitive analytical tools are used [39,40]. Therefore, 800 MHz NMR equipped with cryogenically cooled micro-coil (1.7 mm) probe used in the current study was expected to provide the metabolomic differences between IVF and ICSI derived embryos from non-male factor infertility patients.

To the best of our knowledge, this is the first study to investigate metabolomics signature in human embryos utilizing the state-of-the-art non-invasive and highly sensitive NMR technology. Notably, the use of split IVF/ICSI in this study provided the opportunity to assess metabolomics signatures in the sibling embryos that were able to develop to morphologically optimal blastocyst on day 5.

## Materials and methods

### Patient selection

This prospective study included infertile couples (n = 19) undergoing assisted reproductive treatment at the University infertility clinic between April 2019 to January 2020. Institutional Ethics Committee approval (Kasturba Medical College and Kasturba Hospital Institutional Ethics Committee reference IEC: 237/2019) was obtained before the initiation of the study. Patients who agreed to sign the informed consent and fulfilled the following criteria were included in the study: i) women < 35 years of age with body mass index between 18.5–22.9 kg/m^2^ ii) having regular menstrual cycles without any abnormality/surgery to the reproductive system as disclosed in their medical history; ii) absence of pathological conditions such as endometriosis, tubal abnormalities iii) absence of other metabolic/ endocrine system-associated diseases, such as hypo/hyperthyroidism or hyperprolactinemia iv) the male partners of the patients had semen characteristics above the WHO 2010 reference range [41]. The patient information, including demographic characteristics and data from routine clinical investigations are shown in Table 1.

**Table 1:** Patient’s demographics and clinical characteristics.

Age-female (year± SD)	31.47±2.13
Age-male (year± SD)	37.84±3.53
Duration of infertility (year ± SD)	6.10±3.14
Basal FSH (mIU/mL± SD)	6.36±1.99
Basal LH (mIU/mL± SD)	4.90±1.51
Basal E2 (pg/mL± SD)	37±4.57
AMH (ng/mL± SD)	4.72±2.19
AFC (n± SD)	12.26±5.37
Duration of COS (days ± SD)	10.32±1.37
Estradiol (pg/mL± SD)	4425±1827.69
LH (mIU/mL± SD)	2.45±1.51
Progesterone (ng/mL± SD)	1.13±0.43
Total sperm number (m± SD)	138±68.45
Sperm motility (%±SD)	57.63±15.64
Sperm morphology (% normal forms ± SD)	43.85±7.77
Sperm DNA damage (%±SD)	9.73±2.97

### Terminal Deoxynucleotidyl Transferase dUTP Nick End Labelling (TUNEL) assay

In order to assess the DNA fragmentation, the spermatozoa from each male partner were subjected to TUNEL assay as described earlier with minor modifications [42]. Briefly, sperm cells were fixed on a poly-L-lysine coated coverslip using 4% paraformaldehyde for 30 min. Following the fixation, permeabilization was performed for 30 min using 0.2% Triton X-100. The cells were incubated with terminal deoxynucleotidyl transferase and the nucleotide mix labelled with FITC (Apoalert DNA fragmentation assay kit, Cat No. 630108; Clontech, Mountain View, CA) for 1 hour at 37°C in a humidified chamber. The cells were washed, counterstained with 10 μg/mL propidium iodide and mounted on a glass slide. The TUNEL positive cells were identified by the presence of strong nuclear green fluorescence under a fluorescence microscope (Imager-A1; Zeiss, Gottingen, Germany). A minimum of 1,000 spermatozoa were assessed from each subject and TUNEL index was calculated.

### Controlled ovarian stimulation (COS) and oocyte aspiration

COS was performed using antagonist protocol. Administration of recombinant FSH (rFSH, Gonal F®, Merck Biopharma) was initiated from Day 2 of the cycle. The starting dose of rFSH was 225 to 450 IU/day depending on age, Antimullerian hormone level and antral follicular count. Thereafter, the rFSH dose was increased/decreased depending on the ovarian response until the day prior to hCG administration. Pituitary down-regulation was performed with daily administration of GnRH antagonist (Cetrotide ® 0.25 mg, Merck Biopharma) starting from Day 5 of the stimulation. Oocyte maturation was triggered by the administration of recombinant human chorionic gonadotropin (Ovitrelle® 250 μg, Merck Biopharma) when at least four follicles reached a mean diameter of 18 mm. Oocytes were collected by transvaginal ultrasound-guided follicular aspiration under anesthesia. Oocyte cumulus complexes (OCC) were rinsed and placed in Onestep medium (Vitromed GmbH, Germany; Cat No. V-OSM-20) at 37°C under 6% CO_2_ for 2–3 h.

### Fertilization and embryo assessment

The sibling OCC’s were randomly assigned to split insemination by conventional IVF and ICSI techniques. Briefly, conventional IVF was performed in 80 μL insemination droplet containing 15,000 to 20,000 spermatozoa from the processed fraction of the ejaculate, overlaid with pre-incubated oil (Vitromed GmbH, Germany; Cat No V-OIL-P100). OCC was transferred to each insemination droplet and co-incubated with spermatozoa at 37°C, 6% CO_2_ and 5% O_2_ in MIRI^®^ Multiroom incubator (ESCO Medical, Singapore). Post 16-18h co-incubation, cumulus cells and zona bound spermatozoa were removed entirely by mechanical pipetting and repeated washing steps. Sperm survival was assessed from the insemination droplets using inverted microscope. Subsequently, embryos were transferred individually to freshly prepared 30μL droplet of Onestep medium covered with oil. After fertilization check, embryos were cultured at 37°C, 6% CO_2_ and 5% O_2_ in MIRI^®^ Multiroom incubator.

In ICSI, single sperm was selected under an inverted microscope and injected into the denuded metaphase II oocyte under standard laboratory conditions using Olympus-Narishige workstation. Following injection, oocytes were rinsed and cultured individually in 30 μL droplet of Onestep medium covered with oil. Fertilization assessment was done at 16–18 h post ICSI and embryos were transferred to the freshly prepared 30 μL droplet of Onestep medium and cultured at 37°C, 6% CO_2_ and 5% O_2_ in MIRI^®^ Multiroom incubator.

Embryos from both the groups were assessed for cell number, developmental progression and morphological abnormalities at a regular interval until day 5 of development as per the ESHRE consensus using inverted phase-contrast microscope [43]. Blastocysts were scored as morphologically optimal when they were expanded and had at least B graded inner-cell mass and trophectoderm [43]. On day 5, embryos were either selected for transfer or cryopreservation and 25 μL of the spent culture medium of those that developed into optimal blastocyst was collected carefully without oil contamination and placed individually into labeled sterile cryovials, snap frozen in liquid nitrogen, and then stored at -80°C until used for NMR analysis. Every experimental group (IVF or ICSI) in each patient included one medium droplet without the embryo, maintained at identical conditions, which served as the internal control henceforth referred to as medium control.

### Sperm chromatin dispersion assay

DNA fragmentation in the processed fraction of the spermatozoa and post co-incubation was assessed by sperm chromatin dispersion (SCD) test with minor modifications [44]. Briefly, about 0.1 million spermatozoa were mixed with 1% low melting point agarose and then layered onto a microscopic slide pre-coated with 0.65% normal melting point agarose. Gel on the slides was allowed to solidify followed by denaturation in 0.08 N HCl for 7 min at room temperature in dark. Proteins were removed by exposing the slides to lysis solution I (0.4 M Tris, 20 mM dithiothreitol and 1% SDS pH 7.5) for 20 min. Subsequently, slides were immersed in lysis solution II (0.4 M Tris; 2 M NaCl) for 15 min prior to neutralization in 0.4 M Tris buffer for 2 min. The slides were then dehydrated using ethanol gradients, stained with ethidium bromide (2 μg/mL), and observed under fluorescence microscope (Imager-A1 Carl Zeiss, Gottingen, Germany). A minimum of 500 spermatozoa were scored under 40X magnification from each data point. Spermatozoa with small halo, no halo and fragmented nuclei were considered to calculate the percentage of cells with DNA damage.

### NMR sample preparation and analysis

The spent culture media (SCM) samples were thawed for 10 min at room temperature. 25 μL of each sample was diluted to 35 μL using deuterium oxide (D_2_O) solution containing a pre-calculated amount of TSP (Sodium salt of 2,2,3,3 tetradeutero 3-(trimethyl silyl) propionate) as a standard reference compound and transferred to 1.7 mm NMR tubes. Thus, all the metabolites present in the SCM were diluted upto ~1.4 times with the dilution solution. The dilution solvent was prepared by adding 0.05g of TSP/mL D_2_O and diluting by factor of 10 using D_2_O solvent. This solution (~10 μL) was added to 25 μL culture medium sample to get a working solution containing 8.29 mM of TSP.

NMR experiments were performed on 800 MHz Bruker Advance NMR spectrometer equipped with 1.7 mm cryo-probe at 298 K. One dimensional (1D) ^1^H NMR spectra were obtained using the Carr-Purcell-Meiboom-Gill (CPMG) pulse sequence. CPMG 180 degree pulse train for a duration of 12 ms was used to suppress the protein signals from the media. Each spectrum was obtained using 9615 Hz spectral width, 5s relaxation delay, 16k time domain points, 4 dummy scans and 256 transients.

The time domain data from Free Induction Delay (FID’s) were apodized with a Shifted Sine Bell window function (SSB = 2) and zero-filled to 65536 points prior to Fourier transform. Bruker Topspin version 3.6.2 software was used for NMR data acquisition and processing. A total of 131 1D ^1^H spectra were acquired which included medium control (n = 37) and 47 SCM each from IVF and ICSI group. A total of 12 metabolite peaks were identified based on spectral databases [45,46]. Relative concentrations of the identified metabolites were then obtained by normalizing metabolite peak integrals to the peak integral of the internal standard, TSP.

### Statistical analysis

The clinical and demographic characteristics of the participants enrolled in the study has been represented as mean ± standard deviation (mean±SD). A univariate analysis of the embryological characteristics was implemented using GraphPad InStat 3.0 statistical package. Subsequently, a descriptive analysis of the 12 metabolites across IVF versus ICSI was carried out using CRAN R 3.6.1 [47], where Shapiro-Wilk normality test was used to identify the distribution of data points. Following this, a descriptive comparison of metabolite values between IVF, ICSI and Media Control was also implemented. Metabolic differences between the two insemination techniques were studied by two-dimensional bi-plot [48] obtained as a visualization of the first two Principal Components (PCs; PC1 and PC2) associated with the twelve metabolites captured across 94 samples. The PCs which account for 99.95 of variability in the data; do not appear to clearly differentiate between the two fertilization techniques considered in the study. Further, this observation was validated through a split-plot Analysis of Variance using Satterthwaite’s method [49] at 5% level of significance.

## Results

### Patient characteristics, fertilization outcome and embryo quality

This prospective study included 19 infertile couple who underwent ART treatment. Patient demographics and clinical characteristics are depicted in Table 1. Similarly, embryological observations are provided in Table 2. The average number of oocytes inseminated was comparable between IVF and ICSI. However, the fertilization rate was significantly higher in ICSI group (P< 0.01). The number of spermatozoa survived in the insemination droplet at the end of sperm-OCC co-incubation was 53.94±15.14. Similarly, sperm DNA fragmentation as measured by SCD test did not vary significantly between the processed fraction prior to the insemination (22.27±4.02) and post co-incubation (23.06±4.001). When embryos were assessed for their progression and morphology on day 3, the quality was found comparable. Similarly, day 5 embryo quality was comparable between the groups (Table 2).

**Table 2:** Embryological characteristics.

	IVF	ICSI
Oocytes inseminated (mean ± SD)	13.68±5.27	11.73±4.77
Fertilization rate (%±SD)	56.62±24.15	77.19±16.35*
Day 3- good quality embryos (%±SD)	44.91± 23.79	49.25±25.18
Day 3- fair quality embryos (%±SD)	31.85± 19.51	35.76± 21.91
Day 3- poor quality embryos (%±SD)	16.83±18.46	12.23±18.47
Blastocyst rate (%±SD)	71.52±28.00	75.18±26.14
Day 5- good quality embryos (%±SD)	20.02±21.06	19.51±19.39
Day 5- fair quality embryos (%±SD)	26.28±26.71	35.73 ± 21.44
Day 5- poor quality embryos (%±SD)	21±17.13	17.37±16.24

^*^P<0.*01 with the corresponding group*.

### SCM metabolites in day-5 sibling blastocysts

**Fig 1:**
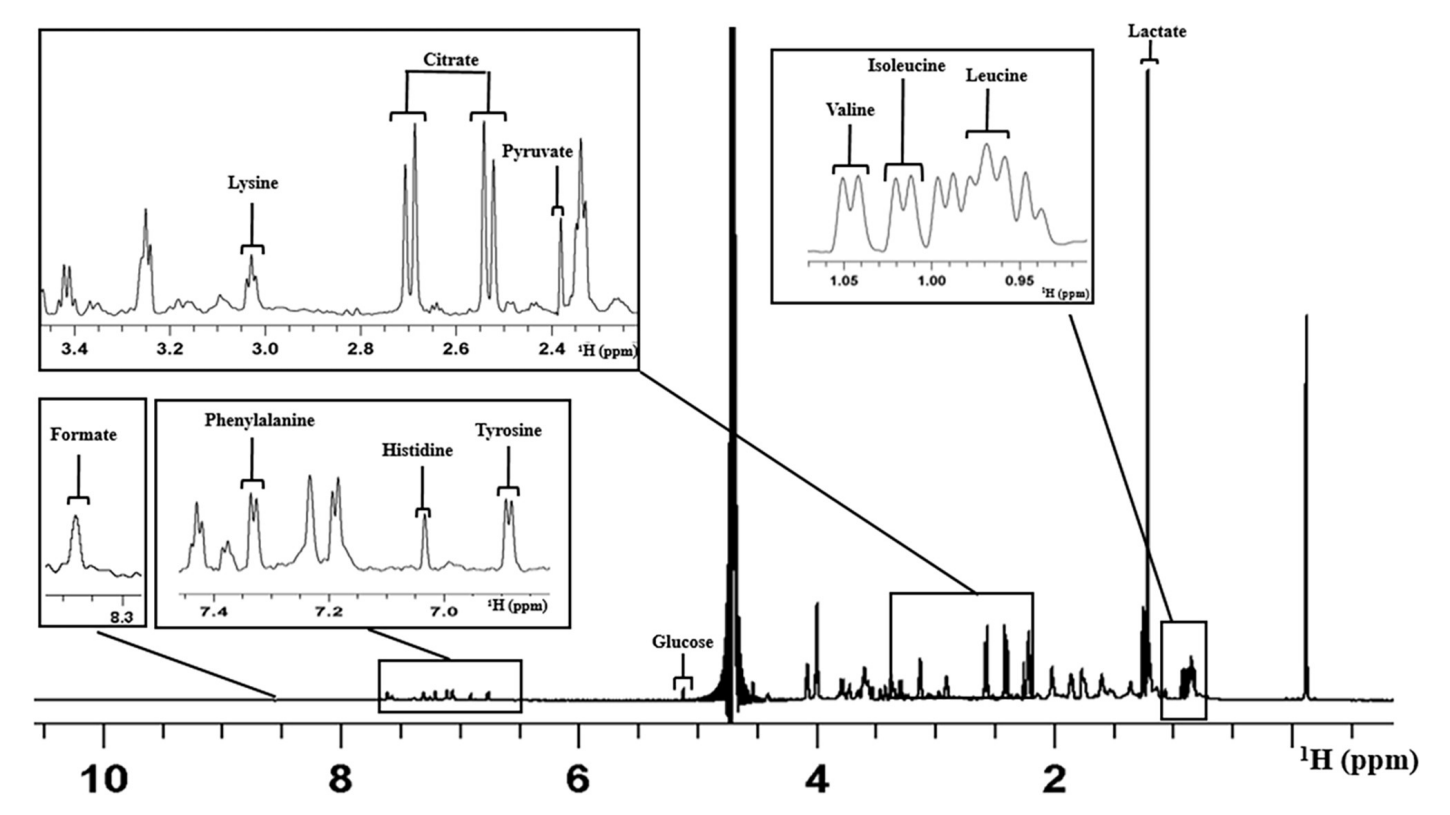
Representative figure of 1D ^1^H NMR spectrum of the one-step embryo culture medium used in this study. The figure elucidates the assignment of peaks for different metabolites. X-axis represents the chemical shift in parts per million.

Metabolomic signature from SCM was assessed in a total of 131 1D proton NMR spectra. Spent media from only good quality blastocysts on day 5 of development as per Istanbul consensus [4] was used to assess the metabolomic signature. The average number of SCM used for metabolomic profiling from both IVF and ICSI groups per patient was approximately 2.5 (from a total of 47 IVF and 47 ICSI derived SCM from 19 patients). In addition, 37 medium control samples were profiled to obtain baseline intensity of metabolites. Fig 1 shows a representative 1D proton NMR spectrum of Onestep medium with the assignment of peaks. A total of 12 metabolites were considered for the analysis as peaks appeared were clear and distinct in all the spectra. This included the amino acid metabolites such as leucine, isoleucine, valine, lysine, tyrosine, histidine and phenyl alanine. Carbohydrate and metabolic intermediates such as pyruvate, glucose, lactate, citrate and formate were also considered.

**Fig 2:**
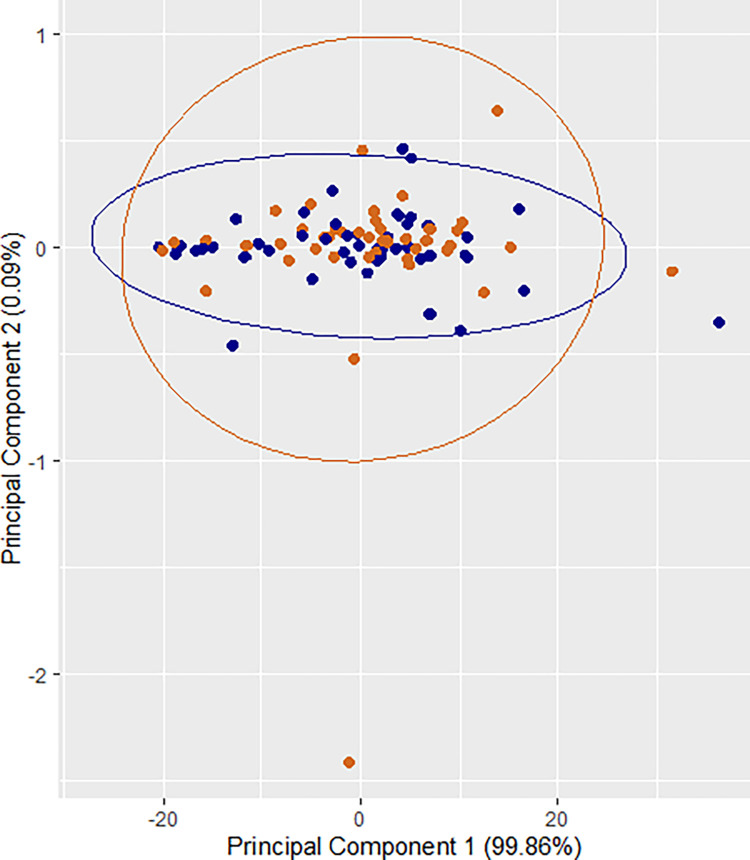
Principal component analysis bi-plot for comparing the relative metabolite intensities of IVF and ICSI derived sibling embryos. PCA plot (PC_1_ vs PC_2_) of SCM demonstrated random distribution of metabolites between IVF and ICSI derived embryos. Each dot represents a single sample. Blue dots (●) represents IVF derived SCM whereas orange dots (●) represents ICSI derived SCM.

Subsequently, relative concentrations of SCM metabolites were compared with the media control to understand the utilization of metabolites from the culture medium. A reduction in metabolite levels was observed for all identified metabolites (Table 3), which indicates that both IVF and ICSI sibling embryos utilized metabolites from the embryo culture medium during the 96 hours of *in vitro* culture. However, only four metabolites from IVF embryos and six metabolites from ICSI embryos demonstrated significant reduction compared to medium control (P< 0.05–0.001; Table 3). Importantly, between IVF and ICSI SCM, there were no significant differences in the levels of the metabolites (Table 3). A multivariate exploration of the 12 metabolites using the two dimensional principal component bi-plots (identified as PC_1_ and PC_2_) did not demonstrate any identifiable differentiation between the metabolite levels of IVF and ICSI derived embryos (Fig 2). Further, a split-plot Analysis of Variance using Satterthwaite’s method on the metabolites confirmed that there is no significant difference in the mean values of metabolites across IVF and ICSI groups (F_1, 56_ = 0.3984, P = 0.5305). This indicates that IVF and ICSI derived embryos are comparable in terms of their metabolite utilization *in vitro* over a period of 96 hours of development.

**Table 3:** Comparison of the relative intensities between IVF and ICSI derived embryo SCM metabolites (normalized to TSP) along with medium control metabolites. Data represented in mean ± SD.

	Intensity (mean ± SD)					
Metabolites	Medium control	IVF (n = 47)	*P* value	ICSI (n = 47)	*P* value	*P* value IVF vs ICSI
Leucine	2.284±2.222	1.776±0.888	0.14	1.795±0.912	0.20	0.91
Isoleucine	1.282±1.151	0.983±0.488	0.07	0.995±0.441	0.07	0.90
Valine	1.335±1.196	1.022±0.508	0.06	1.026±0.449	<0.05	0.96
Pyruvate	0.784±0.748	0.512±0.265	<0.01	0.506±0.232	<0.001	0.90
Citrate	4.147±3.609	2.901±1.477	<0.01	2.931±1.311	<0.01	0.91
Lysine	1.189±1.099	0.818±0.429	<0.01	0.835±0.385	<0.01	0.83
Glucose	0.425±0.391	0.309±0.157	<0.05	0.313±0.146	<0.05	0.89
Tyrosine	0.417±0.375	0.323±0.165	0.11	0.323±0.143	0.06	0.99
Histidine	0.171±0.141	0.128±0.066	0.05	0.126±0.060	<0.05	0.84
Phenyl alanine	0.383±0.310	0.293±0.142	0.08	0.303±0.140	0.08	0.83
Lactate	27.21±23.958	20.95±10.56	0.06	21.32±9.498	0.05	0.86
Formate	0.012±0.024	0.030±0.115	0.30	0.017±0.017	0.54	0.45

### Impact of paternal factors on SCM metabolites

**Fig 3:**
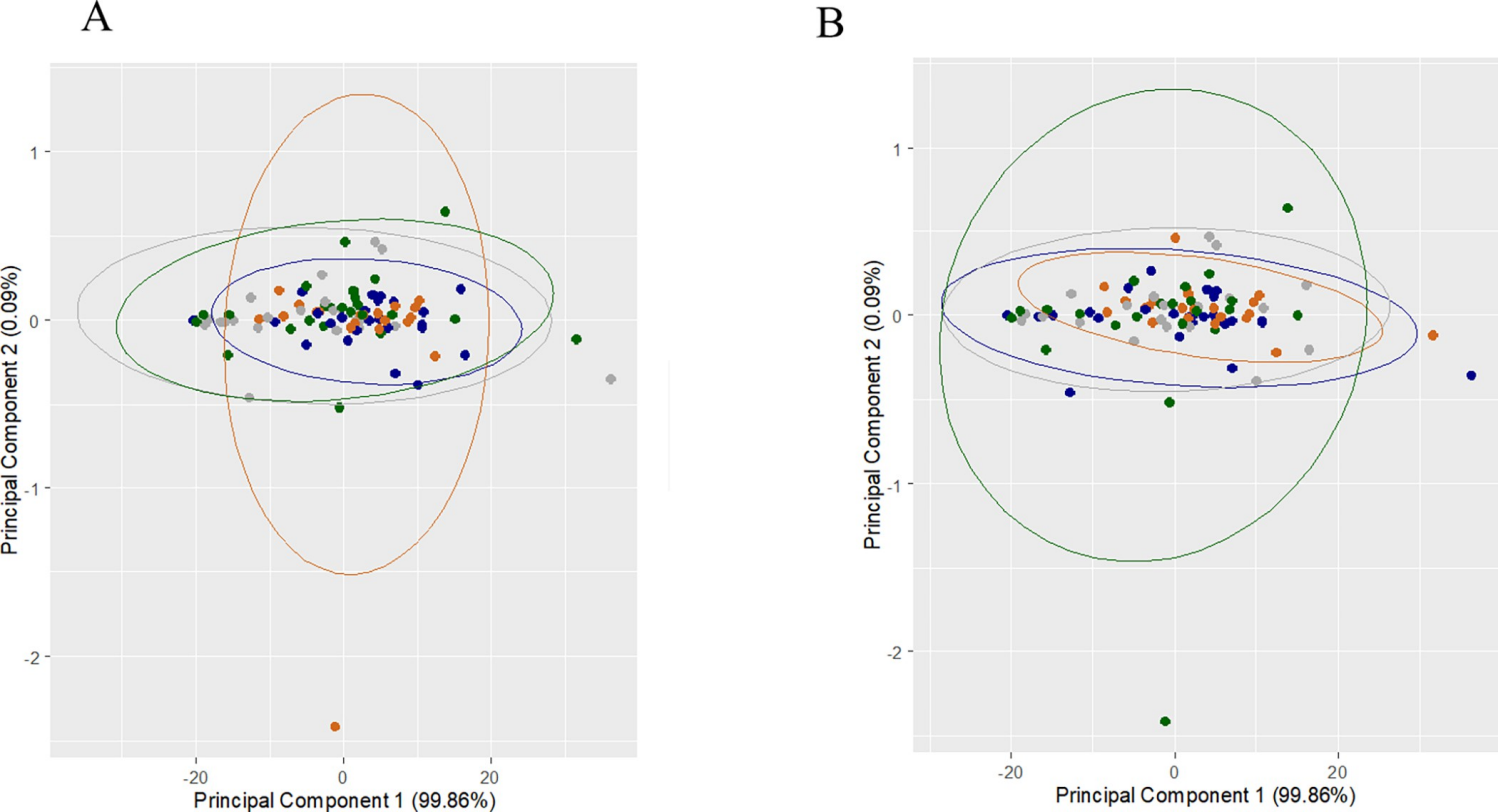
Principal component analysis bi-plot of the relative metabolite intensities of IVF and ICSI derived sibling embryos in relation to paternal factors. A) Bi-plot (PC1 vs PC2) of relative metabolite intensities of IVF and ICSI derived embryos in relation to sperm head defects did not demonstrate differential clustering. Blue dots (●) represents <15% sperm head defects from IVF and orange dots (●) represents >15% sperm head defects from IVF; whereas grey dots (●) represents <15% sperm head defects from ICSI and green dots (●) represents >15% sperm head defects from ICSI. B) Bi-plot (PC1 vs PC2) of relative metabolite intensity of IVF and ICSI embryos in relation to TUNEL index showed random distribution of data points. Blue dots (●) represents <10% TUNEL index from IVF and orange dots (●) represents >10% TUNEL index category from IVF group; grey dots (●) represents <10% TUNEL index and green dots (●) represents >10% TUNEL index category from ICSI group.

Paternal factors have shown to play an important role in embryo development. Though, spermatozoa from only non-male factor patients were used in this study, we made an attempt to look into the impact of specific sperm defects such as head abnormality and DNA fragmentation on SCM metabolites. In the case of sperm head defects, the samples were categorized as patients having <15% (n = 7) and >15% sperm head defects (n = 12). SCM intensities were comparable between IVF and ICSI groups irrespective of sperm head defects (S1 Table). Subsequently, principal component analysis of the metabolites failed to show differential clustering of the data points indicating that the percentage of sperm head defects does not alter SCM metabolites in non-male factor patients (Fig 3A).

Further, metabolite levels were tested to determine any association with sperm DNA fragmentation as measured by the TUNEL assay. Similar to sperm head defects, a comparison was made between the ejaculates having <10% (n = 10) and >10% TUNEL positive sperm (n = 9). It was observed that embryos derived from the ejaculate having <10% DNA fragmented spermatozoa had comparable metabolite levels in both IVF and ICSI group (S2 Table). Similarly, DNA fragmentation >10% did not influence the SCM metabolites both in IVF and ICSI groups (S2 Table). The two-dimensional bi-plot demonstrated the random distribution of data points, clarifying no association of sperm DNA fragmentation on the SCM metabolite intensities of the sibling embryos (Fig 3B).

## Discussion

Existing evidence do not support the preference for ICSI over IVF in non-male factor patients. However, increase in the number of ICSI cycles across the world, controversial rationale for the increase in the number of ICSI cycles and the risks associated with the technique has prompted us to determine whether any metabolomic differences exist in sibling embryos derived from normozoospermic ejaculates. As ICSI bypasses natural selection process, we hypothesized a differential metabolomic signature in this cohort of sibling embryos. However, our data demonstrated comparable level of metabolites in the good quality sibling blastocysts. These observations imply no overall metabolomic differences in the blastocysts derived from non-male factor infertility partners in relation to the insemination techniques used.

Though, fertilization rate was higher in ICSI group, the embryo progression and quality during the preimplantation development was comparable between two groups which is in agreement with the earlier study [33]. On the other hand, studies have also found higher fertilization rates with conventional IVF when compared to ICSI cycles [10,50,51]. These inconsistent and contradictory results could be attributed to the patient cohort used in their studies, pathology and the subjective nature of embryo grading methods employed. However, our results have obtained from the sibling gametes from non-male factor partner, fertilized by two different techniques and then developed *in vitro* for 96 h under identical laboratory conditions.

Metabolomics approach, which involves measurement of many metabolites simultaneously, has the potential to identify biomarkers to predict and improve outcomes in reproductive medicine [52]. Non-invasive assessment of metabolites in the embryo spent medium has enabled monitoring of the developmental progression [53,54]. Further, several metabolic pathways in the preimplantation stage embryos are modulated in response to genetic insults in mammalian cells. Analysis of metabolites in the SCM revealed a significant association between metabolites such as pyruvate, lactate, glucose, proline, lysine, alanine, valine, isoleucine and thymine and the extent of genetic instability observed in the mouse blastocysts [55]. As shown in Table 3, our results have demonstrated a decrease in the level of all twelve metabolites in the SCM in comparison to the media control suggesting the utilization of the substrates from the medium during 96 h *in vitro* culture. Specially, only ICSI-derived blastocysts showed reduced consumption of histidine and valine in comparison to medium controls. At the moment it is not possible to explain the significance of this subtle variation, but definitely warrants further investigation in this direction. However, multivariate analyses demonstrated the lack of relationship between the method of fertilization and the levels of metabolites (Table 3, Fig 2).

Conventional IVF involves prolonged co-incubation of spermatozoa and cumulus-oocyte complexes. Reactive oxygen species (ROS) generated by dead or abnormal spermatozoa may negatively affect the fertilization and subsequent embryonic development. To understand the possible impact of ROS during conventional IVF, we assessed sperm survival soon after the termination of gamete co-incubation. Sperm DNA damage was also assessed prior to the insemination and soon after the termination of sperm-OCC co-incubation. Sperm survival did not vary significantly across the samples. Similarly, sperm DNA damage post-insemination was comparable to pre-insemination level. These observations suggest no significant negative influence of factors during gamete co-incubation on the embryo quality and metabolomic signature.

Paternal genetic alterations may affect embryo viability and reproductive outcomes. It has been shown that paternally transmitted DNA lesions influence the metabolism in preimplantation embryos [31,56]. Low glutamine level was found on day 3 SCM in male factor patients [31]. Since ICSI completely bypasses natural sperm selection, we investigated the differences in the level of metabolites in relation to sperm DNA fragmentation and sperm head defects. Embryos derived from DNA fragmented spermatozoa progressed normally until the blastocyst stage, showed reduced pyruvate-to-alanine ratio in SCM [56]. Hence, we tested the spent medium from the optimal quality blastocyst on day 5. However, a principal component analysis of our data did not demonstrate any association between sperm defects (both DNA fragmentation and head defects) and metabolism in both IVF and ICSI embryos.

A recent study by Leary and Sturmey [33] analyzed the spent embryo metabolites from seven patients and found that neither individual nor collective differences in amino acid metabolism were apparent for sibling oocytes subjected to either IVF or ICSI. On the other hand, from non-sibling cohorts, slightly different amino acid turnover between both groups were observed. Our results showed comparable levels of amino acids in ICSI and IVF in optimal quality blastocysts formed on day 5. Though, we shared a common objective, study outline, and comparable results, the key difference in our study are i) use of spermatozoa from exclusive non-male factor patients ii) bigger sample size iii) profiling of day 5 embryo SCM instead of day 7 in different embryo culture media which might influence the level of metabolites and their stability due to extended culture, and importantly iv) use of sensitivity enhanced NMR as the analytical tool. NMR spectroscopy based metabolomics approach utilized in this study is uniquely suited for the measurement of SCM metabolites non-invasively with no need for sample processing prior to analysis. The use of NMR spectrometer with high frequency (800 MHz) facilitated analysis of SCM metabolites with improved resolution and sensitivity. Further, importantly, the cryogenically cooled micro-coil probe (1.7 mm) provided an extreme boost (>10 fold) to sensitivity. Use of this CryoMicroProbe^TM^ enabled fast NMR data acquisition with more than 200-fold reduction in experiment time. This tool is extremely useful for investigation of metabolism, particularly, using mass limited samples such as the low volume SCM samples as used in this study. To the best of our knowledge, this is the first study to investigate metabolomics signature in human embryos utilizing the state-of-the-art non-invasive and highly sensitive NMR technology.

It has been shown that embryos carrying induced DNA lesions develop normally until blastocyst stage and were morphologically indistinguishable from control embryos [55]. Hence, only morphologically normal embryos were used in this study for the metabolomic analysis. In this way, we could exclude embryos that had problems due to gamete factors or suboptimal laboratory conditions which have the tendency to accelerate metabolic activity during preimplantation development. Further, the metabolic analysis of poorer quality sibling embryos that were not suitable for transfer or cryopreservation is not expected to provide any additional benefit from the clinical perspective.

## Conclusion

The use of sensitivity enhanced NMR spectroscopy to compare the SCM metabolomic signature in sibling blastocysts revealed that in non-male factor infertility patients, the ICSI does not significantly impact on the metabolism of embryos. Though, our hypothesis was not proven in this study, the technique should be utilized for the indication it was developed for, and not as a first-line insemination method for patients with healthy sperm and oocyte. This is a comparison between the metabolomic profiles of day-5 IVF and ICSI blastocysts, among which embryo transfer selection is performed. In this pilot study, we are only interested in whether the insemination method affects blastocyst metabolites. Further research should focus on comparison of the metabolomic profiles of day-5 blastocysts that resulted in pregnancy and those that failed to implant.

## Supporting information

S1 Table Comparison of the relative intensities of embryo SCM metabolites (normalized to TSP) with sperm head defects. (DOCX)

S2 Table Comparison of the relative intensities of SCM metabolites (normalized to TSP) with TUNEL index. (DOCX)

S1 File (XLSX)
