# Supplementary material for: ICSI in non-male factor infertility patients does not alter metabolomic signature in sibling embryos as evidenced by sensitivity enhanced nuclear magnetic resonance (NMR) spectroscopy
Source: PLoS One. 2022 Sep 23;17(9):e0273321. doi: 10.1371/journal.pone.0273321 (PMC9506644; doi:10.1371/journal.pone.0273321)
Supplement: S1 Table — (DOCX) [file pone.0273321.s001.docx]

**S1 Table:** Comparison of the relative intensities of embryo SCM metabolites (normalized to TSP) with sperm head defects

| **Metabolites** | **Relative intensity**  **(mean±SD)** | | ***P* value** | **Relative intensity**  **(mean±SD)** | | ***P* value** |
| --- | --- | --- | --- | --- | --- | --- |
|  | **<15% sperm head defects** | |  | **>15% sperm head defects** | |  |
|  | **IVF**  **(n=25)** | **ICSI**  **(n=21)** |  | **IVF**  **(n=22)** | **ICSI**  **(n=26)** |  |
| Leucine | 2.036±0.066 | 1.851±0.802 | 0.39 | 1.481±1.024 | 1.750±1.006 | 0.36 |
| Isoleucine | 1.136±0.364 | 1.064±0.304 | 0.47 | 0.809±0.556 | 0.939±0.527 | 0.41 |
| Valine | 1.182±0.382 | 1.097±0.312 | 0.41 | 0.840±0.577 | 0.969±0.535 | 0.42 |
| Pyruvate | 0.613±0.201 | 0.555±0.184 | 0.31 | 0.396±0.285 | 0.466±0.261 | 0.38 |
| Citrate | 3.423±1.120 | 3.214±0.964 | 0.50 | 2.309±1.628 | 2.703±1.515 | 0.39 |
| Lysine | 0.972±0.341 | 0.924±0.287 | 0.61 | 0.643±0.458 | 0.764±0.442 | 0.35 |
| Glucose | 0.360±0.118 | 0.337±0.103 | 0.49 | 0.250±0.177 | 0.293±0.173 | 0.40 |
| Tyrosine | 0.376±0.125 | 0.343±0.098 | 0.34 | 0.264±0.186 | 0.307±0.171 | 0.40 |
| Histidine | 0.151±0.051 | 0.132±0.048 | 0.21 | 0.102±0.072 | 0.120±0.068 | 0.38 |
| Phenyl alanine | 0.336±0.110 | 0.315±0.099 | 0.51 | 0.245±0.160 | 0.292±0.167 | 0.33 |
| Lactate | 24.24±7.921 | 22.93±6.686 | 0.55 | 17.21±12.048 | 20.02±11.238 | 0.40 |
| Formate | 0.045±0.157 | 0.015±0.006 | 0.38 | 0.012±0.009 | 0.019±0.022 | 0.23 |
