# Supplementary material for: ICSI in non-male factor infertility patients does not alter metabolomic signature in sibling embryos as evidenced by sensitivity enhanced nuclear magnetic resonance (NMR) spectroscopy
Source: PLoS One. 2022 Sep 23;17(9):e0273321. doi: 10.1371/journal.pone.0273321 (PMC9506644; doi:10.1371/journal.pone.0273321)
Supplement: S2 Table — (DOCX) [file pone.0273321.s002.docx]

**S2 Table**: Comparison of the relative intensities of SCM metabolites (normalized to TSP) with TUNEL index

| **Metabolites** | **Relative intensity (mean±SD)** | | ***P* value** | **Relative intensity**  **(mean±SD)** | | ***P* value** |
| --- | --- | --- | --- | --- | --- | --- |
|  | **<10% TUNEL index** | |  | **>10% TUNEL index** | |  |
|  | **IVF**  **(n=26)** | **ICSI**  **(n=23)** |  | **IVF**  **(n=21)** | **ICSI**  **(n=24)** |  |
| Leucine | 1.811±0.911 | 2.097±0.671 | 0.22 | 1.733±0.879 | 1.506±1.028 | 0.435 |
| Isoleucine | 1.011±0.502 | 1.125±0.361 | 0.37 | 0.948±0.479 | 0.870±0.482 | 0.59 |
| Valine | 1.042±0.518 | 1.157±0.368 | 0.38 | 0.997±0.507 | 0.901±0.491 | 0.52 |
| Pyruvate | 0.531±0.275 | 0.571±0.212 | 0.57 | 0.488±0.257 | 0.443±0.237 | 0.55 |
| Citrate | 2.993±1.519 | 3.344±1.122 | 0.36 | 2.789±1.451 | 2.536±1.377 | 0.55 |
| Lysine | 0.874±0.444 | 0.961±0.334 | 0.44 | 0.748±0.409 | 0.715±0.399 | 0.79 |
| Glucose | 0.328±0.166 | 0.365±0.120 | 0.39 | 0.284±0.145 | 0.263±0.154 | 0.63 |
| Tyrosine | 0.331±0.170 | 0.364±0.118 | 0.43 | 0.314±0.161 | 0.284±0.156 | 0.53 |
| Histidine | 0.131±0.066 | 0.139±0.054 | 0.66 | 0.124±0.067 | 0.113±0.063 | 0.55 |
| Phenyl alanine | 0.304±0.144 | 0.335±0.127 | 0.42 | 0.280±0.141 | 0.271±0.147 | 0.83 |
| Lactate | 21.62±11.029 | 24.215±8.392 | 0.36 | 20.12±10.160 | 18.55±9.832 | 0.60 |
| Formate | 0.044±0.154 | 0.016±0.007 | 0.39 | 0.013±0.007 | 0.018±0.023 | 0.33 |
